# Bacteriology of the conjunctiva in pre-cataract surgery patients with occluded nasolacrimal ducts and the operation outcomes in Japanese patients

**DOI:** 10.1186/s12886-017-0410-x

**Published:** 2017-02-20

**Authors:** Yuko Hayashi, Takeshi Miyamoto, Shuko Fujita, Katsuo Tomoyose, Nobuyuki Ishikawa, Masahide Kokado, Takayoshi Sumioka, Yuka Okada, Shizuya Saika

**Affiliations:** 1 Department of Ophthalmology, Wakayama Medical University, 811-1 Kimiidera, Wakayama, 641-0012 Japan; 2 Department of Ophthalmology, Wakayama Medical University Kihoku Hospital, 219 Myoji, Katsuragi-cho, Itogun, Wakayama, 649-7113 Japan

**Keywords:** Nasolacrimal duct obstruction, Conjunctiva, Bacterium, Cataract surgery

## Abstract

**Background:**

Contamination of the conjunctiva in association with nasolacrimal duct obstruction is by all accounts a risk factor for infectious endophthalmitis post-cataract surgery.

**Methods:**

All patients who underwent cataract day surgery routinely received nasolacrimal duct syringing with normal saline at the Wakayama Medical University Hospital, Japan, from 2011 to 2013. The microorganisms isolated from conjunctival swab samples of patients with occluded nasolacrimal ducts and their susceptibility to antibiotics, as well as the operation outcomes in all the patients were retrospectively investigated.

**Results:**

Nasolacrimal duct obstruction was observed in 125 eyes of 90 patients (3.3%; 42 eyes of 30 male individuals, and 83 eyes of 60 female individuals) from a total of 3754 eyes of 2384 patients by using irrigation samples of nasolacrimal ducts. The mean age of the subjects with duct obstruction was 79 ± 8.5 years.. In bacterial cultures of swabs from these 125 individuals, microbial growth was detected in 56 samples (i.e. 44.8%). Coagulase-negative *Staphylococcus* was detected in 28 eyes, and *Corynebacterium* species was detected in 17 eyes. *Staphylococcus aureus*, excluding methicillin-resistant *S. aureus* was detected in seven eyes with nasolacrimal duct obstruction. Methicillin-resistant *S. aureus* was isolated in two eyes with nasolacrimal duct obstruction. Each case was treated with topical antibiotics based on the results of antibiotic sensitivity tests. After culturing of cotton swab samples from the conjunctiva, and using direct micrography of bacteria every 2 or 3 days after starting treatment, and once the results were negative (consecutively tested three times), the patients received cataract surgery. In the current case series, bacteria were not detected in conjunctival swabs obtained consecutively three times for 3 weeks after starting topical antibiotics in 118 eyes from 125 eyes (94.4%), and later in the remaining patients. No patient required dacryocystorhinostomy to eliminate bacterial contamination in the conjunctiva following topical antibiotic therapy. No patient developed infectious endophthalmitis at least 1-year post-cataract surgery.

**Conclusions:**

All the patients receiving cataract day surgery underwent the operation after the elimination of conjunctival microorganism contamination in association with nasolacrimal duct obstruction by using appropriate topical antibiotics.

## Background

Infectious endophthalmitis occurs in 0.04–0.075% of patients following cataract surgery [1–3]. Colonies of endogenous normal bacterial flora are the main source of bacterial contamination in the anterior chamber of the eye. *Staphylococci*, *Enterococci*, or Gram-negative bacilli could cause poor visual prognoses postinfection, although the incidence is reportedly low [4–7]. Gram negative bacteria reportedly constitute an increasing proportion of the bacteria found in chronic dacryocystitis and they may be a reservoir for postoperative intraocular infection [8]. It was suggested that these bacteria account for higher rates of nasolacrimal duct obstruction among patients who developed infectious endophthalmitis after cataract surgery [9–11]. Furthermore, these studies indicated that nasolacrimal duct obstruction might cause lacrimal sac and conjunctival bacterial contamination even in the absence of dacryocystitis. Such an occurrence could also be a intraocular infection risk factor subsequent to either trauma or surgery.

It is beneficial, therefore, to test for the presence of nasolacrimal duct obstruction prior to cataract surgery to prevent postoperative bacterial infection in the eye. We routinely perform syringing of the nasolacrimal duct in pre-cataract surgery patients. Furthermore, in all the patients having nasolacrimal duct obstruction regardless of the presence or absence of dacryocystitis we examine both their washings and conjunctival swab samples for bacteriological growth. The susceptibilities of the bacteria isolated from the conjunctival swab samples of patients with occluded nasolacrimal duct, to antibiotics, were determined to eliminate conjunctival bacterial contamination. In the current retrospective study, none of the patients received surgical treatment i.e. tubing or dacryocystorhinostomy, for nasolacrimal duct obstruction at our institution to eliminate bacterial contamination in conjunctiva. The present study retrospectively summarizes our results and the outcomes of cataract surgery during 3 years (2011–2013) along with those abovementioned cases receiving pre-surgical antibiotic treatment. As a result of being given suitable topical antibiotics prior to nasolacrimal duct obstruction surgery, even though those individuals had positive bacterial cultures, there were no cases of postoperative endophthalmitis.

## Methods

### Patients

**Fig. 1:**
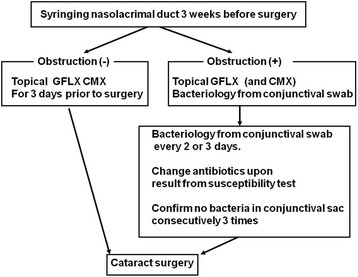
Examination of the nasolacrimal duct and disinfection of the conjunctiva prior to cataract surgery. GFLX: gatifloxacin CMX: cefmenoxime

This retrospective study was approved by the institutional committee for clinical research All the patients undergoing day surgery for cataract (3754 eyes of 2384 patients) received syringing (irrigation) of the nasolacrimal duct with normal saline at Wakayama Medical University Hospital, Wakayama, Japan, 3 weeks prior to the operation due date, for a 3-year period from 2011 to 2013. Bacteriological examinations and/or antibiotic susceptibility testing of samples isolated from conjunctival swabs were performed in patients with obstruction of the duct prior to surgery. Bacteria-positive patients received further examination of conjunctival swab samples at every 2 or 3 days, along with topical administration of antibiotics that were effective for the detected microorganism (Fig. 1). The patients underwent cataract surgery after obtaining negative results for bacterial infection consecutively for three times. Phacoemulsification and aspiration of the crystallin lens with implantation of an intraocular lens (IOL) was performed in 3707 eyes (98.7%) and planned extracapsular cataract extraction with IOL implantation was performed in 47 eyes (1.3%). No patient developed infectious endophthalmitis post-cataract surgery.

### Bacteriological examinations

Conjunctival swab samples were obtained from patients with nasolacrimal duct obstruction by using cotton swabs under local anesthesia with topical lidocaine. On the same day, bacteriological culturing (blood agar medium, chocolate agar medium, MacConkey’s medium, Colombian nutrient medium, and *Candida* medium) was conducted. Each culture was incubated in 35 °C for 16–20 h. Anaerobic culturing was not performed. The minimum inhibitory concentration was classified as “S” (susceptible) or “R” (resistant) by employing the criteria developed by the Clinical and Laboratory Institute.

All patients with nasolacrimal duct obstruction had received topical gatifloxacin 0.3%(GFLX) before receiving bacteriological examinations as described above. Patients with positive bacterial cultures received further administration of topical cefmenoxime hydrochloride0.5% (CMX) when the detected bacteria were susceptible to these antibiotics. All patients with detected microorganisms by culture examination after detecting nasolacrimal duct obstruction received further conjunctival swab examinations every 2 or 3 days along with topical antibiotics as necessary. Cataract surgery was performed after obtaining no bacterial positive results from the conjunctival swab cultures consecutively for three times.

Prior to the surgery, the eyelid skin and conjunctival sac were disinfected with povidone-iodine. The patients received intravenous sodium piperacillin during or immediately after the surgery. The affected eye received levofloxacin (LVFX) ointment after the operation. From the next day, the patients received topical GFLX for 1–3 months and oral cefcapene pivoxil hydrochloride for 4 days.

## Results

### The incidence of nasolacrimal duct obstruction

Nasolacrimal duct obstruction was observed in 125 eyes of 90 patients (3.3%; 42 eyes of 30 male individuals, and 83 eyes of 30 female individuals) in a total of 3754 eyes of 2384 patients. The mean age of the subjects with duct obstruction was 79 ± 8.5 years.

### Bacteriological examinations of conjunctiva with nasolacrimal duct obstruction

Bacteriological cultures detected microbial growth from the swab samples of 56 of 125 subjects from 90 patients at the initial bacterial culturing (44.8%). Percentages for Gram-positive cocci, Gram-positive rods, Gram-negative bacilli, and fungi were 64, 24, 11, and 1%, respectively (Table 1). Among 125 eyes with positive results for bacteria or fungi, coagulase-negative *Staphylococcus* (CNS), *Corynebacterium* species, or *S. aureus* were detected in 28, 17, and seven eyes, respectively. Methicillin-resistant *S. aureus* (MRSA) was observed in two of seven eyes with *S. aureus*.

**Table 1:** Bacteria and fungus detected at the first conjunctival swab culture in patients with nasolacrimal duct obstruction

Gram-positive cocci	Coagulase-negative Staphylococci	28
	Staphylococcus aureus (including MRSA in 2 eyes)	7
	Enterococcus facalis	3
	Streptococcus speices	6
Gram-positive bacilli	Corynebacterium species	17
Gram-negative bacilli	Enterobacter	3
	Moruganella	2
	Klebsiella	2
	Proteus	1
Fungus	Candida albicans	1

*MRSA* methicillin-resistant Staphylococcus aureus

### Bacterial results following antibiotic treatment prior to surgery

As shown in Table 2, detected microorganisms exhibited resistance to antibiotics. The majority of the bacteria detected were found to be resistant to LVFX, cefazolin (CEZ), and/or gentamycin.

**Table 2:** Sensitivity of detected microorganisms and their resistance to each antibiotic

Bacterium species	Antibiotic	Resistant	Sensitive
Coagulase-negative Staphylococci	LVFX	5	17
	CEZ	5	15
	GM	4	15
MSSA	LVFX	1	5
	CEZ	0	6
	GM	1	5
MRSA	LVFX	3	0
	CEZ	3	0
	GM	2	1
Streptococcus species	LVFX	1	2
	CEZ	1	1
Corynebacterium species	LVFX	3	2
	CEZ	0	4
Gram-negative bacilli	LVFX	1	7
	CEZ	3	5
	GM	1	7

*MSSA* methicillin-susceptible Staphylococcus aureus

*MRSA* methicillin-resistant Staphylococcus aureus

The majority of the bacteria were found to be resistant to levofloxacin (LVFX), cefazolin (CEZ), or gentamycin (GM)

Table 3 includes all the cases in the current series that required additional topical administration of antibiotic eye drops besides the new quinolone drugs and cephem antibiotics. Conjunctival bacterial contamination was successfully eliminated by administration of topical dibekacin0.3%(DKB)or arbekacin0.5%(ABK)sulfate in case 1 or in cases 2 and 3, respectively. In three eyes of two patients, MRSA was detected at the second or later examination, but not at the first examination. MRSA detected in this case series exhibited resistance to LVFX and CEZ, but was sensitive to vancomycin(VCM). In case 7, *Corynebacterium* appeared after Gram-negative bacilli were eliminated with topical GFLX0.3%. Because this occurred during topical quinolone drug administration, the *Corynebacterium* was eliminated with topical tobramycin0.3%(TOB). In the current case series, no microorganisms were detected in conjunctival swabs consecutively for three times at 3 weeks after starting topical antibiotics in 118 eyes of 125 eyes (94.4%). Durations of more than 3 weeks were required before no bacteria were detected in swab cultures from conjunctiva taken consecutively for three times in cases 4–7 (Table 3).

**Table 3:** Cases that required administration of additional topical antibiotic eye drops in addition to quinolone and cephem drugs to achieve a negative result for bacterial infection

Case #	Bacterium species	Sensitivity	Additional antibiotic
1	CNS	LVFX (R) CEZ (R) GM (R) ABK (S)	DKB
2	CNS	LVFX (R) CEZ (R) GM (R) ABK (S)	ABK
3	CNS	LVFX (R) CEZ (R) GM (R) ABK (S)	ABK
4	MRSA	LVFX (R) CEZ (R) GM (R) VCM (S)	VCM
5	MRSA	LVFX (R) CEZ (R) GM (R) VCM (S)	VCM
6	MRSA Gram-negative bacilli	LVFX (R) CEZ (R) GM (R) VCM (S)	VCM
		LVFX (S) CEZ (S) GM (S)	
7	Corynebacterium Spp. Gram- negative bacilli	LVFX (S) CEZ (S) GM (S)	TOB

*CNS* coagulase-negative Staphylococci

*MSSA* methicillin-susceptible Staphylococcus aureus

*MRSA* methicillin-resistant Staphylococcus aureus

DKB: dibekacin0.3%; ABK; arbekacin0.5%; VCM; vancomycin; TOB: tobramycin

## Discussion

Obstruction of the nasolacrimal duct causes retention of tears in the lacrimal and conjunctival sac, leading to the acceleration of growth of the microorganisms in the accumulated tears. The likelihood of post-cataract surgery infectious endophthalmitis is much higher in patients whose nasolacrimal ducts are obstructed [9, 10]. This association suggests that this condition could be a risk factor for intraocular infection subsequent to either trauma or surgery. In addition, 82% of the bacteria detected from eyes with postoperative infectious endophthalmitis were reportedly genetically identical to those in the normal conjunctival flora [11]. There is no published guideline available in Japan describing a recommended strategy for treating patients with nasolacrimal duct obstruction and have contaminated conjunctivae prior to intraocular surgery. Therefore, examination of the nasolacrimal duct by syringing and bacteriological examinations in eyes with obstruction of the duct should be performed prior to intraocular surgeries according to our protocol.

A previous study found the incidence of adult nasolacrimal duct obstruction to be 3.1%. Anatomical differences may account for the difference in the incidence of obstruction between male and female individuals [12]. Microorganisms were detected in 44.8% of the eyes with nasolacrimal duct obstruction. We did not perform anaerobic culturing, and this could account for the difference in the detection rate (30–80%) between our study and the previous studies [13–16].

CNS was the most prevalent finding, and therefore in the current case series, *Corynebacterium* and *S. aureus* were detected. This result is similar to that of a previous study, although we did not perform anaerobic culturing and could not detect *Propionibacterium acnes *[8]. CNS is the bacterial type most frequently observed in post-cataract surgery infectious endophthalmitis [4]. Obstruction of the nasolacrimal duct reportedly increases the percentage of Gram-negative bacilli in the conjunctiva in non-Japanese subjects [8, 16]. Bacteriology of conjunctiva in healthy Japanese subjects without nasolacrimal duct obstruction was also reported [17, 18]. Suto et al. described bacterial presence without screening for nasolacrimal duct obstruction in pre-cataract surgery patients [14]. Thus this data is not applicable to this study. Hoshi et al. reported an increased incidence of Gram-negative bacilli in Japanese subjects as compared with healthy subjects [17]. Moreover, Omatsu et al. reported that the incidence of Gram-negative bacilli was 3.2% [18]. In the current study, 6.4% of the bacilli were Gram-negative in the conjunctiva among all subjects, which is indicative of a higher incidence than previously reported. As Gram-negative bacilli are one of the notable bacterial groups that are post-cataract surgery pathogens causing endophthalmitis, these findings support the notion that nasolacrimal duct obstruction could be a risk factor of this infection.

Although *Corynebacterium* species is one of the most frequently detected bacterial species in normal bacterial flora in human conjunctiva, it was reported that it causes infection in corneas treated with topical corticosteroid therapy after penetrating keratoplasty [19]. Postoperative infectious endophthalmitis is quite rare, presumably because the aqueous humor contains low levels of lipid components that are required for *Corynebacterium* species to grow. *Corynebacterium* in the conjunctiva is sensitive to cephem antibiotics and aminoglycosides, while the bacterium is frequently resistant to new quinolones [6]. We successfully eliminated *Corynebacterium* that newly appeared after the elimination of Gram-negative bacilli by adding the aminoglycoside in case 7. The phenomenon observed in case 7 was considered to be a form of microbial substitution.

## Conclusions

In summary, nasolacrimal duct obstruction was observed in 125 eyes of 90 patients (3.3%; 42 eyes of 30 male individuals, and 83 eyes of 60 female individuals) from a total of 3754 eyes of 2384 patients by using samples from syringing of the nasolacrimal duct with normal saline. Each case with positive microorganisms cultured from conjunctival swabs was treated with topical antibiotics based on the results of susceptibility tests. After results from culturing of cotton swab samples from the conjunctiva, and by direct micrography for bacteria every 2 or 3 days (consecutively for three times) were negative, the patients received cataract surgery. Normalization of bacterial flora in the conjunctiva with nasolacrimal duct obstruction reportedly requires 4–5 weeks after dacryocystorhinostomy [20]. Another report showed that dacryocystorhinostomy decreased the percentage of conjunctival sac Gram positive cases from 82% to 36% [21] We successfully eliminated bacterial contamination of the conjunctiva in 94.4% of the patients with nasolacrimal duct obstruction at 3 weeks before cataract surgery, and eventually also eliminated bacterial contamination in all the other cases. In our series, no patient required tubing or dacryocystorhinostomy to eliminate bacterial contamination in the conjunctiva following topical antibiotic therapy. No patient developed infectious endophthalmitis for at least 1-year post-cataract surgery who received topical antibiotic treatment, although previous reports already suggested that nasolacrimal duct obstruction is a potential risk factor of post-cataract surgery infectious endopthalmitis.

## Abbreviations

ABK: Arbekacin
CEZ: Cefazolin
CMX: Cefmenoxime
CNS: Coagulase-negative *Staphylococcus*
DKB: Dibekacin
GFLX: Gatifloxacin
IOL: Intraocular lens
LVFX: Levofloxacin
MRSA: methicillin-resistant *Staphylococcus aureus*
MSSA: Methicillin-susceptible *Staphylococcus aureus*
TOB: Tobramycin
VCM: Vancomycin
